# A Rare Case of Bilateral Pheochromocytomas: Diagnostic and Surgical Challenges in a 38-Year-Old Female

**DOI:** 10.7759/cureus.85885

**Published:** 2025-06-12

**Authors:** Jawad Khan, Malik Furqan Mahmood, Mudassar Abbas, Aleena Ejaz, Eman Riaz

**Affiliations:** 1 Urology, Ayub Teaching Hospital, Abbottabad, PAK; 2 Acute Medicine, St. George's University Hospital, London, GBR; 3 Radiology, Ayub Teaching Hospital, Abbottabad, PAK; 4 Surgery, Ayub Medical College, Abbottabad, PAK

**Keywords:** adrenal surgery, adrenal tumors, bilateral pheochromocytoma, catecholamine-secreting tumor, cortical-sparing adrenalectomy, hypertension, multidisciplinary approach, staged adrenalectomy

## Abstract

Pheochromocytomas are rare tumors of the adrenal medulla, and bilateral involvement is especially uncommon in the absence of a hereditary syndrome. We present the case of a 38-year-old female who presented with bilateral flank pain, excessive sweating, and palpitations. Clinical examination and laboratory investigations revealed elevated blood pressure and markedly increased serum metanephrines and normetanephrines. Imaging identified large bilateral adrenal masses with features suggestive of pheochromocytomas. A multidisciplinary team opted for a staged surgical approach: a left adrenalectomy followed by a right cortical-sparing adrenalectomy. The patient was adequately prepared preoperatively with alpha- and beta-blockers to reduce the risk of intraoperative hypertensive crisis. Histopathology confirmed pheochromocytoma in both adrenal glands. Postoperative management included steroid replacement therapy and regular monitoring of adrenal hormone levels to assess the functionality of the remaining adrenal tissue. Genetic testing was offered to assess for underlying genetic causes, but was declined by the patient. This case illustrates the importance of individualized, multidisciplinary care in managing bilateral pheochromocytomas, with emphasis on preserving adrenal function and ensuring long-term surveillance.

## Introduction

Pheochromocytomas are tumors that develop from the adrenal medulla's chromaffin cells, which produce, store, metabolize, and typically but not always secrete catecholamines. Paragangliomas are tumors that arise from extra-adrenal chromafín cells. They can come from parasympathetically associated chromaffin tissue (head and neck) or sympathetically associated chromaffin tissue (primarily belly and pelvis, less frequently thorax). Often hormonally active, sympathetic paragangliomas are also referred to as extra-adrenal pheochromocytomas [1]. A migraine-like headache, intermittent or persistent arterial hypertension, sweating, palpitations, anxiety, tremor, nausea, weakness, pallor, weight loss, or postural hypotension are the most typical symptoms [2]. The incidence of pheochromocytoma, a rare tumour, is two to eight cases per million annually. These tumours are challenging to identify and treat, particularly when they affect both sides, even though they account for less than 0.2% of all instances of hypertension. Bilateral pheochromocytomas are extremely uncommon (7% to 10% of pheochromocytomas); a germline mutation is present in 60% to 90% of patients with bilateral tumours [3]. In certain studies, long-term postoperative outcomes have been favourable, with survival rates comparable to those of the general population. Both synchronous and metachronous presentations of bilateral pheochromocytomas are possible. It has been demonstrated that patients with von Hippel-Lindau disease (VHL; VHL gene), multiple endocrine neoplasia type 2 (MEN 2), and paragangliomas syndromes types 1 and 4, which are caused by mutations in the succinate dehydrogenase (SDH) subunit D (SDHD) and B (SDHB) genes, respectively, are more likely to develop bilateral pheochromocytomas, which are frequently heritable [4].

Either periods of excessive catecholamine secretion or chronic hypertension in patients with undiscovered pheochromocytoma are linked to the cardiovascular symptoms of pheochromocytoma. Hypertensive crises are typically linked to severe cardiovascular consequences in pheochromocytoma [5]. Malignancies involving both adrenal glands may provide special endocrinological and surgical challenges. This situation must be carefully thought through to remove the tumour entirely and lower the risk of irreversible adrenal insufficiency. To diagnose and treat dual pheochromocytomas in an adult female, this case emphasises the importance of genetic counselling and interdisciplinary teamwork.

## Case presentation

A 38-year-old female presented with bilateral flank pain, excessive sweating, and palpitations. Upon examination, she had elevated blood pressure and excessive palmar sweating. Laboratory tests revealed significantly elevated serum metanephrines and normetanephrines, indicating catecholamine hypersecretion.

**Figure 1:**
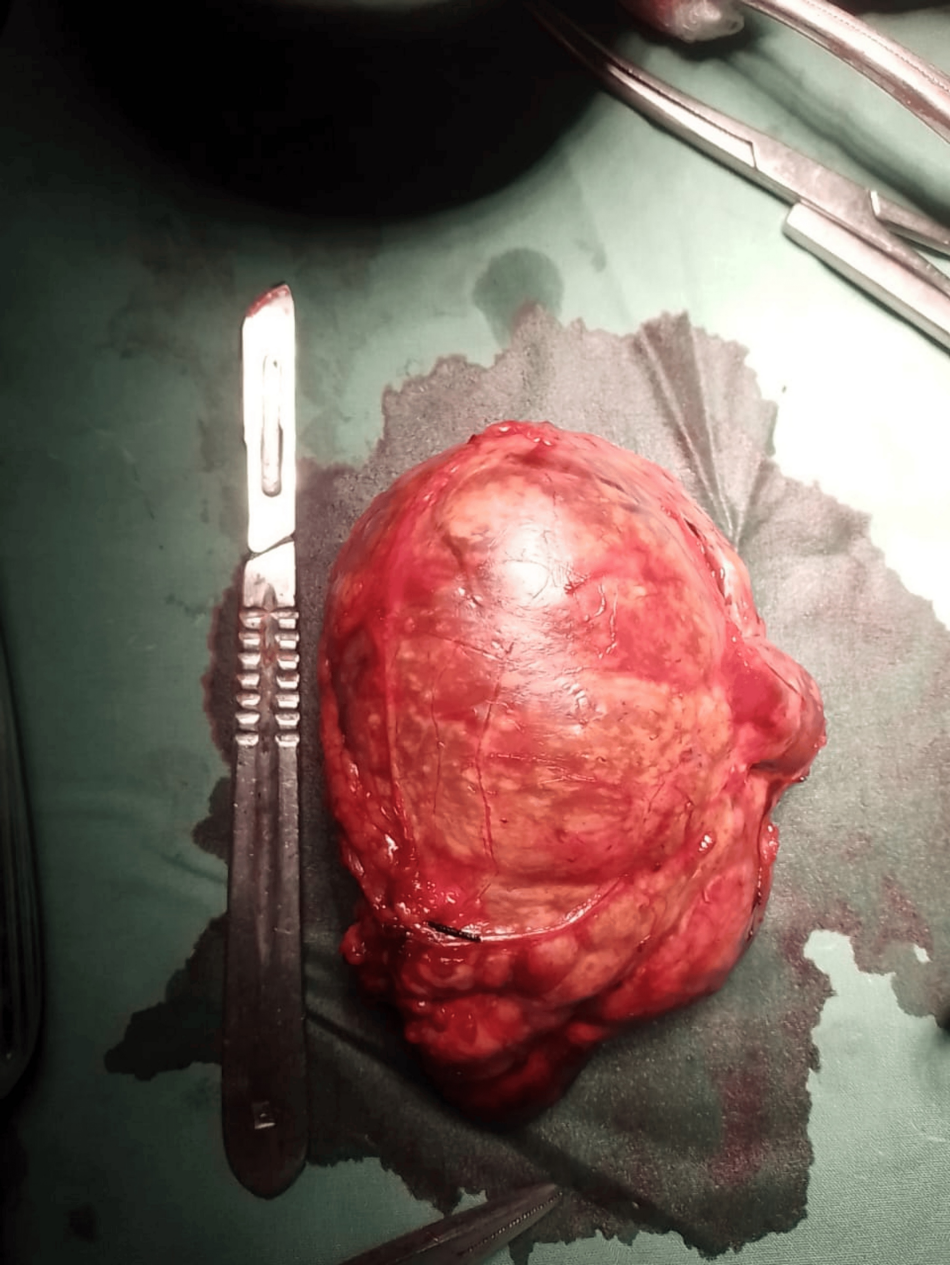
Gross specimen of the resected right adrenal pheochromocytoma The tumor appears encapsulated with a smooth external surface, areas of hemorrhage, and yellowish discoloration; consistent with its vascular and lipid-rich nature.

**Figure 2:**
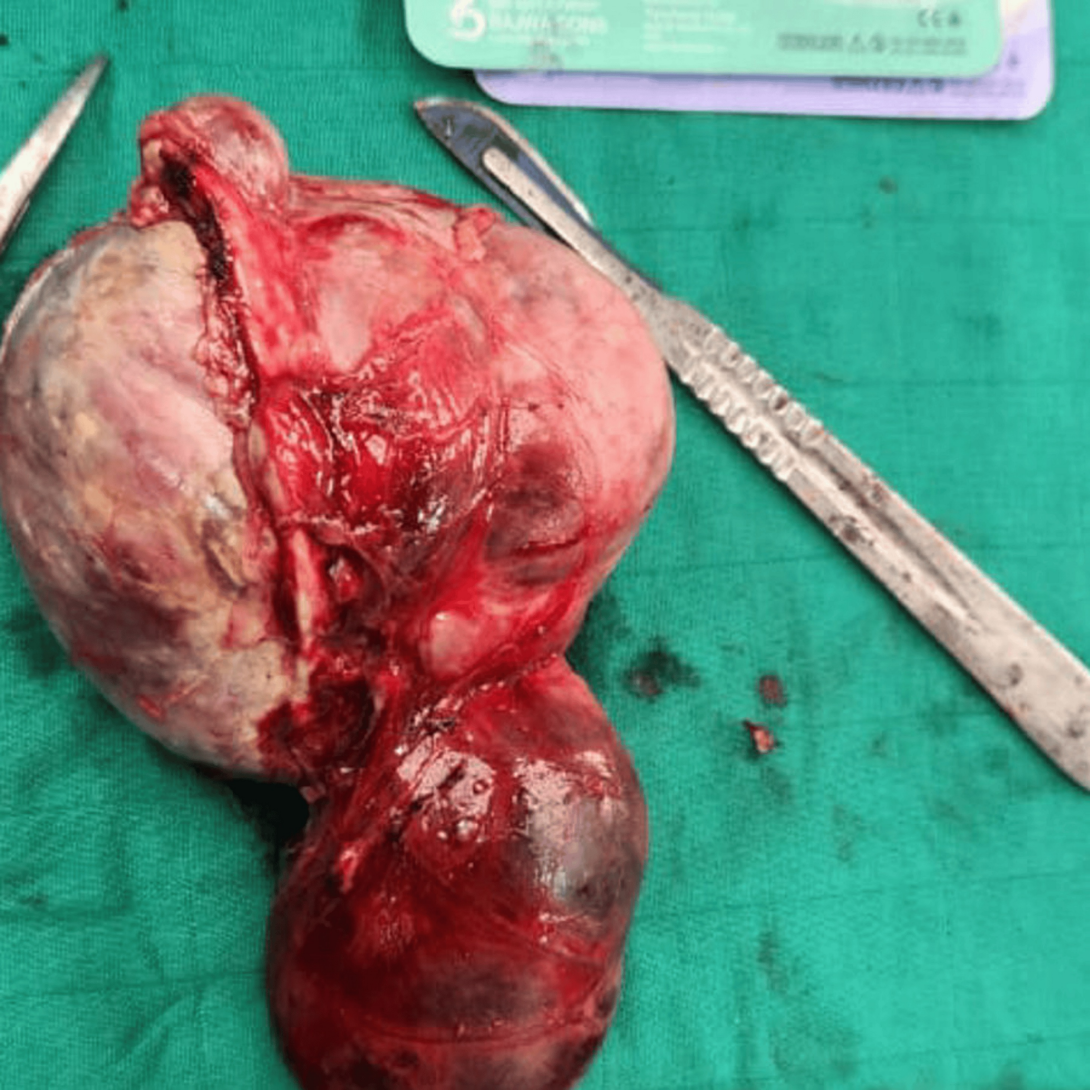
Gross specimen of the resected left adrenal pheochromocytoma The mass appears well-circumscribed and encapsulated, with heterogeneous areas of hemorrhage and congestion on the external surface, consistent with the tumor's vascular nature.

**Figure 3:**
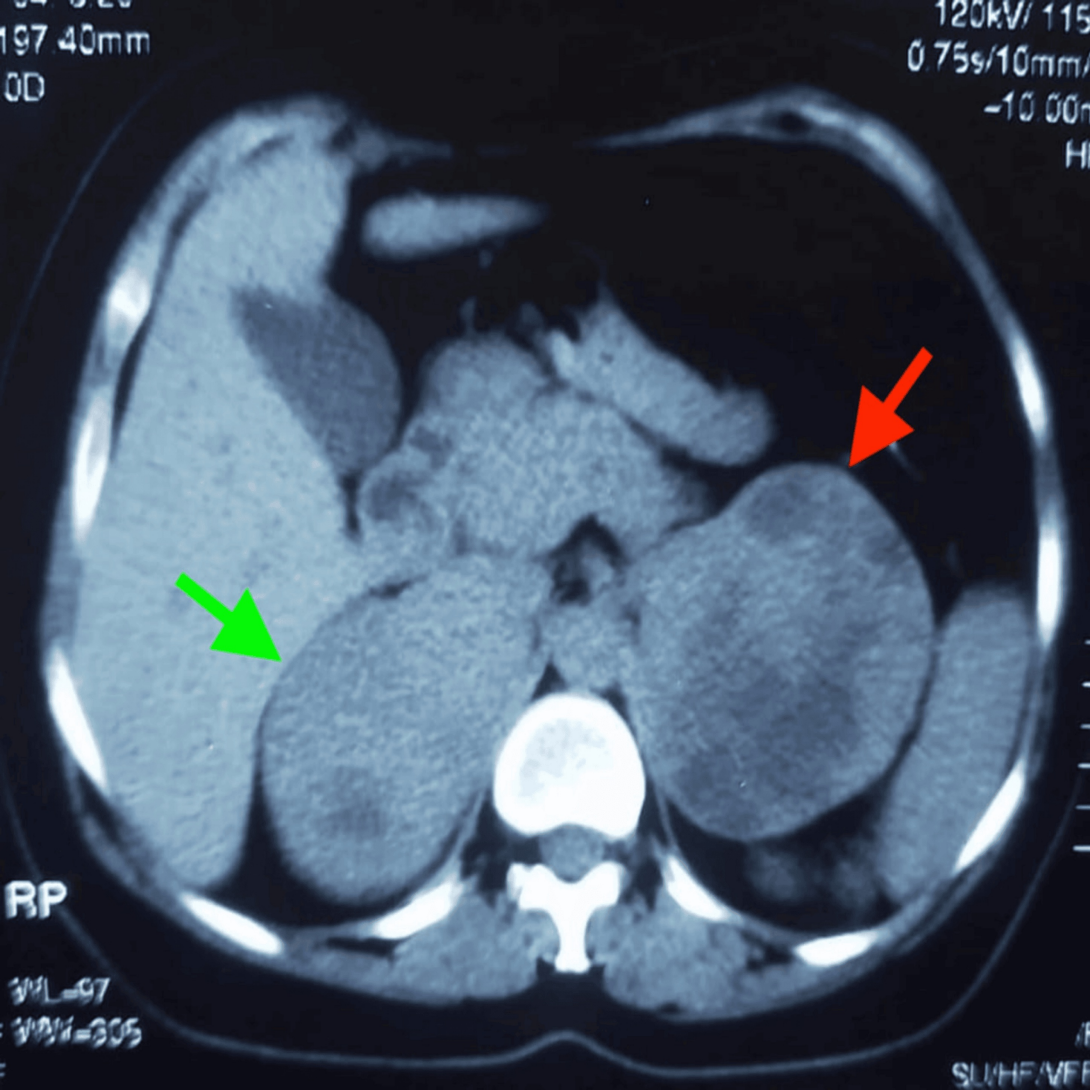
Axial contrast-enhanced CT Scan The scan shows bilateral adrenal masses consistent with pheochromocytomas. The right adrenal mass (green arrow) appears well-defined and heterogeneously enhancing, while the left adrenal mass (red arrow) shows similar enhancement characteristics. No evidence of local invasion or distant metastasis is seen.

Contrast-enhanced CT imaging revealed bilateral adrenal masses (Figure 1). The right adrenal mass (Figure 2) measured approximately 9 cm × 8 cm × 5 cm, and the left adrenal mass (Figure 3) measured approximately 9 cm × 8 cm × 11 cm. Both masses were solid and cystic, with interface loss in the adjacent kidney and spleen. Laboratory investigations showed significantly raised serum metanephrines and normetanephrine.

A multidisciplinary team meeting was convened to discuss the case. A staged surgical approach was chosen, starting with a left adrenalectomy followed by a right cortical-sparing adrenalectomy to preserve adrenal function. The patient was given alpha-adrenergic blockade with phenoxybenzamine to prevent hypertensive crises as preoperative preparation, followed by beta-blockers to manage tachycardia.

**Figure 4:**
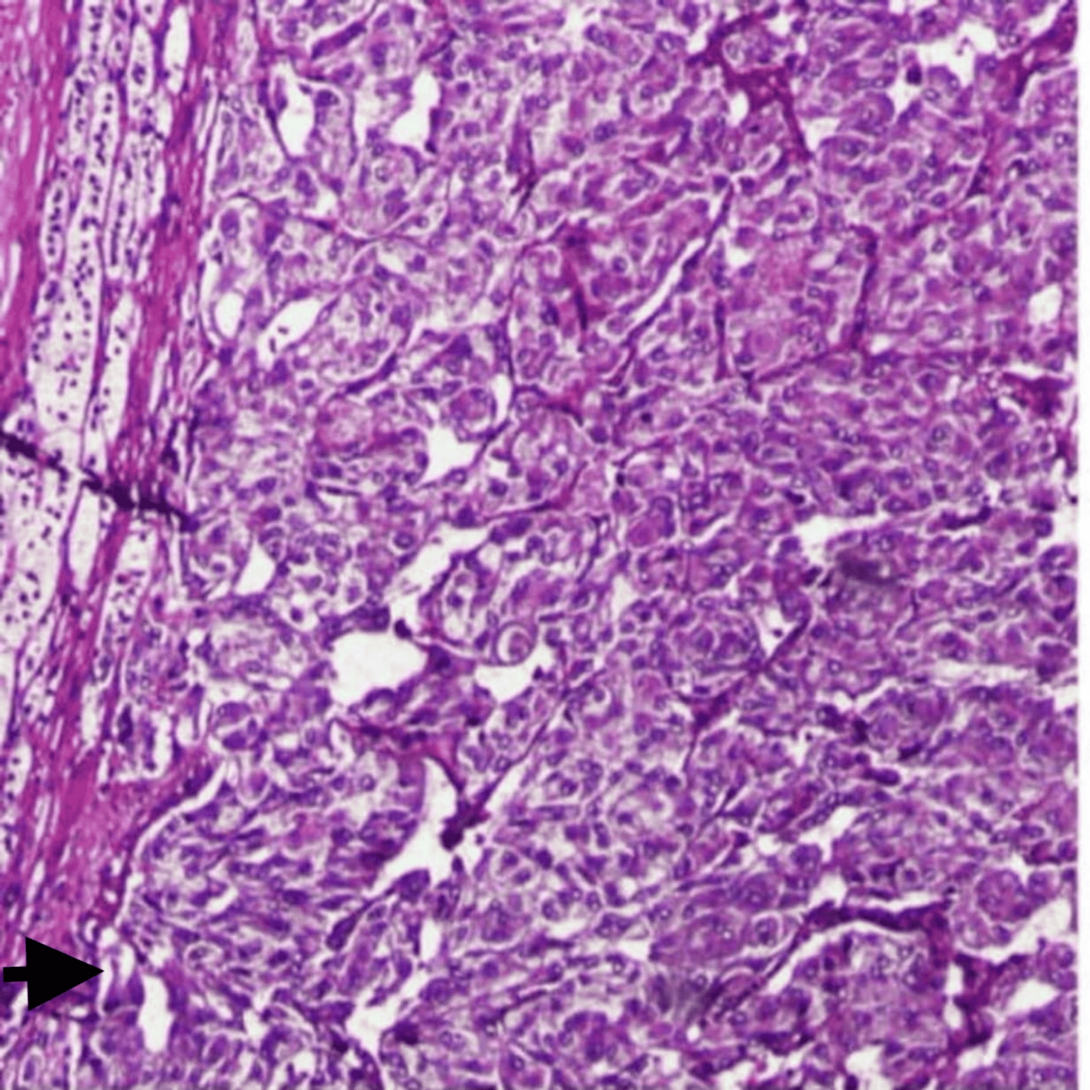
Histopathology image showing pheochromocytoma The H&E magnification (20x) shows a nested pattern of large polyclonal cells with abundant cytoplasm and uniform nuclei (black arrow). H&E: Hematoxylin and eosin

**Figure 5:**
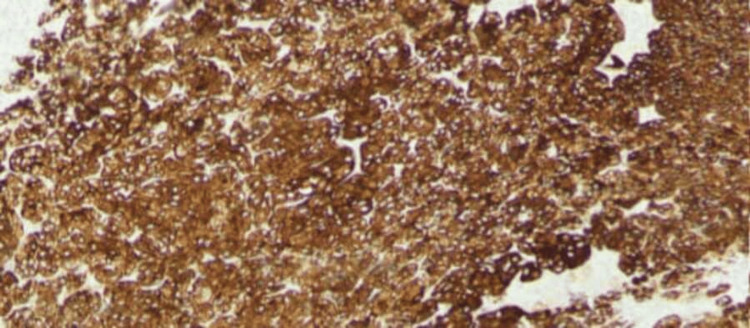
Immunohistochemical staining showing diffuse cytoplasmic positivity The IHC stain synaptophysin is diffusely positive in neoplastic cells (H&E magnification, 20x). IHC: Immunohistochemical, H&E: Hematoxylin and eosin

The left adrenalectomy was uneventful, and histopathology confirmed the diagnosis of pheochromocytoma. Despite continued alpha- and beta-blockade therapy, repeat serum catecholamines remained elevated two months post-surgery, indicating residual functional tumor activity. A right cortical-sparing adrenalectomy was performed two and a half months after the first surgery, and histopathology confirmed another pheochromocytoma (Figures 4-5).

The patient was placed on steroid replacement therapy to manage potential adrenal insufficiency postoperatively. Serum cortisol and adrenal hormone levels were monitored to assess the functionality of the preserved cortex. Genetic testing was offered, but the patient declined. 

## Discussion

Although the existence of a pheochromocytoma can nearly always be confirmed or disproved using current diagnostic techniques, a strong index of suspicion is the key to better identification of an endocrine tumor. All patients with chronic or paroxysmal hypertension should be evaluated for pheochromocytoma, particularly those who exhibit symptoms. These variations in catecholamine release and content, and how they interact with adrenoceptors, account for various clinical manifestations and ultimately call for tailored care for every patient. In these circumstances, medical management can typically control the issue, allowing for the elective removal of the pheochromocytoma.

Typically, a systematic diagnostic approach begins with biochemical testing, such as assessing 24-hour urine catecholamines or plasma-free metanephrines [6]. Plouin and Gimenez-Roqueplo state that the measurement of plasma or urine metanephrines is the primary method for obtaining a positive diagnosis of pheochromocytoma since it is more sensitive than measuring either urine vanillylmandelic acid or plasma catecholamines [7]. Palliative care is currently the most common treatment for metastatic pheochromocytoma. Although surgery has the potential to be curative, the likelihood of a successful resection is reduced by tumor dispersion. Cytoreductive methods, radiopharmaceuticals, chemotherapy, radiation, and experimental therapies are other treatment approaches. Systemic treatment may involve targeted irradiation with I-meta-iodo-benzyl-guanidine (MIBG) (Azedra®). Analogues of radiolabeled somatostatin are being studied [8].

Subtotal adrenalectomy, which involves leaving a small portion of well-vascularized cortical tissue in place following tumor excision, is preferred by several centers to maintain adrenal cortical functions and prevent lifelong steroid replacement treatment. Advocates use the minimal risk of cancer in hereditary pheochromocytomas and the predicted occurrence of bilateral disease to support orthotopic adrenal cortex preservation [9]. Except in cases of extremely early or limited disease, a negative result for plasma free fractionated metanephrines is useful in ruling out paragangliomas and pheochromocytomas. Although the research varies, 24-hour urine total fractionated metanephrines are usually thought to have a somewhat lower sensitivity and a greater specificity than plasma fractionated metanephrines. There is disagreement over whether urine or plasma should be used for testing, and institutional and regional variations exist in the suggested testing methodologies [10]. Our example highlights the importance of a multidisciplinary approach to patient diagnosis, surgery planning, and postoperative care, in addition to contributing to this small but expanding body of information.

The pheochromocytoma surgical operation should only be performed by skilled anaesthesiologists and surgeons. The laparoscopic method is favoured for tumour access, except for situations of probable metastases and tumour size greater than 7 cm, conditions in which the standard open access is required. An ICU should ideally be used for the entire immediate postoperative period since, even with proper preparation, there is a risk of arrhythmias and blood pressure instability, as well as the potential for hypotension and hypertensive crises during this time [11]. More thorough data is required to provide standardised protocols for the diagnosis, surgical decision-making, histopathological evaluation, and long-term management of bilateral pheochromocytomas.

## Conclusions

This case demonstrates the difficulties in diagnosing and treating bilateral pheochromocytomas, an uncommon and possibly fatal endocrine condition. To provide complete care, our experience highlights the value of a multidisciplinary approach combining endocrinologists, surgeons, genetic counselors, radiologists, and pathologists. The patient's good prognosis was largely due to the utilization of cortical-sparing adrenalectomy, meticulous surgical planning, and preoperative alpha-adrenergic inhibition. These tactics reduced the possibility of intraoperative hemodynamic instability and prevented the need for steroids for the rest of the patient's life.

Furthermore, this case report emphasizes the significance of genetic testing in the treatment of bilateral pheochromocytoma, despite its occasionally delicate nature. Finding a genetic condition can help guide surgical strategies and enable early identification and prevention for family members who are at risk. However, it is crucial to respect the autonomy of the patient when it comes to genetic testing.

Lifelong monitoring with biochemical tests and imaging is essential due to the risk of recurrence and the emergence of metachronous malignancies. Further and more extensive documentation of bilateral pheochromocytoma is required to improve clinical awareness and aid in the creation of standardized diagnostic and treatment standards. Optimizing the patient’s quality of life and survival requires careful follow-up, customized surgery planning, and early diagnosis.
